# Stroke risk in older British men: Comparing performance of stroke-specific and composite-CVD risk prediction tools

**DOI:** 10.1016/j.pmedr.2022.102098

**Published:** 2022-12-24

**Authors:** Ayesha Ahmed, Gareth Ambler, Snehal M. Pinto Pereira, Lucy Lennon, Olia Papacosta, Peter Whincup, Goya Wannamethee

**Affiliations:** a Department of Primary Care and Population Health, University College London, UK; b Department of Statistical Science, University College London, UK; c Division of Surgery and Interventional Science, University College London, UK; d Population Health Research Institute, St George’s, University of London, UK

**Keywords:** AF, atrial fibrillation, BRHS, British Regional Heart Study, CHD, coronary heart disease, CIF, cumulative incidence function, CPI, centred prognostic index, CVD, cardiovascular disease, FSRP, Framingham stroke risk profile, HF, heart failure, KM, Kaplan-Meier, MI, myocardial infarction, NICE, National Institute For Health And Care Excellence, PCE, pooled cohort equations, PI, prognostic index, SCORE, systematic coronary risk evaluation, Sn/Sp, percent sensitivity/percent specificity, TIA, transient ischemic attack, Calibration, Cardiovascular disease, Discrimination, Older adults, Risk prediction, Stroke

## Abstract

Stroke risk is currently estimated as part of the composite risk of cardiovascular disease (CVD). We investigated if composite-CVD risk prediction tools QRISK3 and Pooled Cohort Equations-PCE, derived from middle-aged adults, are as good as stroke-specific Framingham Stroke Risk Profile-FSRP and QStroke for capturing the true risk of stroke in older adults. External validation for 10y stroke outcomes was performed in men (60-79y) of the British Regional Heart Study. Discrimination and calibration were assessed in separate validation samples (FSRP n = 3762, QStroke n = 3376, QRISK3 n = 2669 and PCE n = 3047) with/without adjustment for competing risks. Sensitivity/specificity were examined using observed and clinically recommended thresholds. Performance of FSRP, QStroke and QRISK3 was further compared head-to-head in 2441 men free of a range of CVD, including across age-groups. Observed 10y risk (/1000PY) ranged from 6.8 (hard strokes) to 11 (strokes/transient ischemic attacks). All tools discriminated weakly, C-indices 0.63–0.66. FSRP and QStroke overestimated risk at higher predicted probabilities. QRISK3 and PCE showed reasonable calibration overall with minor mis-estimations across the risk range. Performance worsened on adjusting for competing non-stroke deaths. However, in men without CVD, QRISK3 displayed relatively better calibration for stroke events, even after adjustment for competing deaths, including in oldest men. All tools displayed similar sensitivity (63–73 %) and specificity (52–54 %) using observed risks as cut-offs. When QRISK3 and PCE were evaluated using thresholds for CVD prevention, sensitivity for stroke events was 99 %, with false positive rate 97 % suggesting existing intervention thresholds may need to be re-examined to reflect age-related stroke burden.

## 1 Introduction

Population ageing continues to be associated with rising burden of stroke (Feigin et al., 2021, Johnson et al., 2019). Current practice assesses stroke risk together with that of coronary heart disease (CHD) as the composite risk of cardiovascular disease (CVD), through tools such as QRISK3 in England (Hippisley-Cox et al., 2017), Pooled Cohort Equations in the US (PCE) (Goff et al., 2014a) and SCORE2 across Europe (Hageman et al., 2021). There are two main concerns with this approach. Firstly, evidence points to attenuation (Odden et al., 2014) and even reversal (Ahmadi et al., 2015) with increasing age of associations between traditional risk factors and CVD (van Bussel et al., 2020), including stroke (Lind et al., 2018). However, except for the recent SCORE2-OP (SCORE2-OP Working Group and ESC Cardiovascular Risk Collaboration, 2021), development samples for CVD risk tools have been predominantly middle-aged (Bambrick et al., 2016). Secondly, stroke and CHD have interrelated yet distinct pathophysiology. Literature suggests that the relative role and predictive power of conventional risk factors likely differs between heart disease and stroke (Endres et al., 2011, Giang et al., 2013, Syed et al., 2012). Moreover, underlying causes of stroke (Lindley, 2018) and the proportion of heart and circulatory diseases constituted by fatal stroke events (British Heart Foundation, 2022), change with ageing. There is little evidence on how well composite-CVD prediction rules, derived from mostly middle-aged adults, capture the true risk of *stroke* events in *older* adults.

Few stroke-specific risk tools have been validated in an older UK population. The Framingham Stroke Risk Profile (FSRP) (D'Agostino et al., 1994) overestimated risk in European older adults with only average discrimination, particularly among men (Bineau et al., 2009, Voko et al., 2004). QStroke developed later from UK primary care data (Hippisley-Cox et al., 2013) has not been independently validated in older British adults free of prevalent CVD.

To address these research gaps, we first externally validated 2 S-specific (FSRP and QStroke) and 2 composite-CVD (QRISK3 and PCE) risk tools for predicting the 10y risk of stroke outcomes in older men of the British Regional Heart Study (BRHS). Because competing causes of death in older cohorts can affect model performance (Livingstone et al., 2021, Nanna et al., 2020, Nguyen et al., 2020), we considered our external validation with and without adjustment for competing non-stroke mortality. Second, we evaluated how the tools classified men with respect to stroke events using cut-offs based on observed risk, and for composite tools, clinically recommended thresholds for CVD intervention. Finally, we additionally assessed performance of risk tools head-to-head in a common subsample of men who at baseline were free of a wide range of cardiovascular conditions and not under specific CVD prevention treatments, to better inform primary prevention.

## 2 Methods

We follow Transparent Reporting of a multivariable prediction model for Individual Prognosis Or Diagnosis - TRIPOD guidelines for reporting validation studies (Collins et al., 2015).

### 2.1 Summary of development cohorts

#### 2.1.1 FSRP for men

Male participants (n = 2372) of the Framingham Heart Study 55-84y (mean 65y), free of stroke, from examination cycles 9 (1964) and 14 (1975), with 10 years of follow-up (D'Agostino et al., 1994, Wolf et al., 1991). Primary outcome included strokes (ischemic and haemorrhagic) and transient ischemic attacks (TIAs).

#### 2.1.2 QStroke for men

Male patients (n = 1748108) 25-84y (mean 45y) registered on the QResearch primary care database over 1st Jan 1998 – 1st Aug 2012; and without with a history of stroke/TIA and anticoagulant use (Hippisley-Cox et al., 2013). Primary outcome was the first recorded diagnosis of stroke or TIA, excluding haemorrhagic stroke.

#### 2.1.3 QRISK3 for men

Men (n = 3869847) 25-84y (mean 43y), registered on the QResearch database over 1st Jan 1998 – 31st Dec 2015, without history of CVD and statin use. Primary outcome was a composite of CHD, ischemic stroke, and TIA (Hippisley-Cox et al., 2017).

#### 2.1.4 PCE for men

White men (n = 9098) 40-79y (mean 56y), from 4 large US community-based cohorts, free of previous myocardial infarction (MI-recognized or unrecognized), stroke, congestive heart failure (HF), percutaneous coronary intervention, coronary bypass surgery, or atrial fibrillation (AF) with ≥ 12y follow-up (Goff et al., 2014b). Primary outcome was a composite of CHD death, non-fatal MI, fatal and non-fatal stroke.

### 2.2 The BRHS validation sample

BRHS is a prospective study which began in 1978–1980 by recruiting a socially representative sample of 7735 men aged 40-59y, drawn at random from age-sex registers of 24 primary care practices across Britain (Walker et al., 2004). In 1998–2000 (baseline for this analysis), 4252 men 60-79y (mean 68y) participated in the 20y questionnaire-based, physical and clinical re-examination. Follow-up for incident fatal and non-fatal events is available to 2018 through national mortality and 2-yearly primary care record reviews for 96 % of the participants. For external validations, men were followed from baseline to the first of stroke/TIA event or death; or a maximum of 10y to match the time horizon of the above-described risk tools. All participants provided written informed consent in accordance with the Declaration of Helsinki. Ethical approval was obtained from National Research Ethics Service Committee London – Central, Reference number: MREC/02/2/91.

Definitions of endpoints and predictors of all risk tools with corresponding BRHS measures are detailed in Supplementary Tables 1 and 2.

### 2.3 Statistical analysis

We first validated each tool for its respective stroke outcome using sub-samples of men selected per its eligibility criteria. We subsequently examined the FSRP, QStroke and QRISK3, that share ischemic strokes and TIAs as common outcomes, head-to-head in a further common sub-sample of men without a history of stroke, TIA, CHD (MI, angina, percutaneous transthoracic coronary angioplasty and, coronary artery bypass grafting), HF, AF, intermittent claudication and statin or anticoagulant use. We did not include PCE in this sub-analysis because TIAs are not part of its original outcome, which would lead to inherent miscalibration.

Missing data in validation samples ranged from 6 to 12 %, with minimal differences between men with/without complete information, especially with respect to outcome events (Supp. Tables 3A-E). We hence limited our analysis to complete cases. Validation samples fulfilled a minimum of 100 events as criteria for sample size (Collins et al., 2016).

External validation was informed by guidelines from Royston and Altman (Royston and Altman, 2013) and Steyerberg (Steyerberg, 2019), and conducted in Stata 17.

The 4 risk tools model their predictors using (Cox) proportional hazards models. We calculated **10 yr predicted probabilities (P)** of the outcome using

P=1-BaselineS(10)∧exp(CentredprognosticIndex)

where

BaselineS(10) = published 10y baseline survivor function of the relevant risk tool.
Prognostic Index (PI) = linear predictor calculated using published predictor coefficients and BRHS values of predictor variables.
Centred (CPI) = PI centred using published means.

For composite-CVD tools, predicted probabilities were multiplied by the proportion of all events that were stroke/TIA, 0.366 for QRISK3 (calculated from published data (Hippisley-Cox et al., 2017)), or hard strokes, 0.289 for PCE (requested from authors) as analysed elsewhere (D'Agostino et al., 2008, Majed et al., 2013).

**Discrimination** refers to how well a model separates participants who go on to have an event from those that don’t (Royston and Altman, 2013). We assessed this using Harrell’s C index [95 %CI] (somersd package), which can range from 0.5 (as good as chance) to 1 (perfect discrimination). We also visually inspected separation of Kaplan-Meier (KM) survival curves of 4 risk groups according to 16th 50th and 84th centiles of the PI (Royston and Altman, 2013).

**Calibration** refers to the accuracy of a model’s predictions i.e. how closely predicted probabilities agree with observed probabilities, overall and at various levels of predicted risk (Royston and Altman, 2013). We used the beta coefficient of the CPI as a single predictor in a Cox proportional hazards model to measure the **calibration slope [95 %CI]** indicating overfitting where the slope is < 1 and underfitting where the slope is > 1 (Van Calster et al., 2016). We assessed **mean calibration** (calibration in the large) as ratio of global mean predicted risk to observed risk (KM method), where a ratio greater/<1 indicates global over/under-estimation. We assessed **moderate calibration (**Van Calster et al., 2016**)** by comparing KM observed risk at 10y with mean predicted risk in deciles of predicted risk (pmcalplot package (Ensor et al., 2018)), and additionally across 4 age-groups (≤65, >65-≤70, >70-≤75 and > 75 years) in the common subsample.

We examined sensitivity/specificity (Sn/Sp%) of tools using stroccurve package to account for censoring (Cattaneo et al., 2017), at a threshold corresponding to the overall KM observed risk and at conventional clinical thresholds for composite-CVD risk i.e. 10 % for QRISK3 (National Institute for Health and Care Excellence, 2014) and 7.5 % for PCE (Arnett et al., 2019, Goff et al., 2014a).

Sensitivity analyses accounting for competing non-stroke mortality were run as described by Wolber’s et al (Wolbers et al., 2009). Further details available in supplementary methods.

## 3 Results

Comparisons of baseline and performance characteristics between the BRHS validation sample and development sample of each tool are given in Supp. Tables 4–7. Overall BRHS men had a mean age of 68y, and a median follow-up of 10y. A greater percentage of BRHS men were on blood pressure treatment and fewer of them were current smokers. Information on Townsend scores, valvular heart disease (except that indicated by use of anticoagulants and so excluded), systemic lupus erythematosus and mental illness was not available in BRHS.

Table 1 provides summary performance indicators of each validation. There was no violation of proportional hazards over time.

**Table 1:** External validation of stroke-specific and composite-CVD risk tools in older men of the BRHS.

Risk Tool	FSRP		QStroke		QRISK3		PCE	
Sample free of	Stroke, TIA		Stroke, TIA, Anticoagulant use		Stroke, TIA, Coronary Heart Disease, Statin use		Stroke, Myocardial Infarction, Heart Failure, Atrial Fibrillation	
No. of participants	3762		3376		2669		3047	
Events (N)	366 Strokes, TIAs		307 Ischemic Strokes, TIAs		232 Ischemic Strokes, TIAs		184 Strokes	
Validation	Main	CR	Main	CR	Main	CR	Main	CR
Competing Events (N)	–	900	–	784	–	570	–	673
Harrell’s C [95 % CI]	0.6346 [0.6068–0.6624]	0.6155 [0.5876–0.6434]	0.6431 [0.6136–0.6726]	0.6199 [0.5904–0.6495]	0.6317 [0.5970–0.6664]	0.6148 [0.5800–0.6496]	0.6606 [0.6215–0.6997]	0.6422 [0.6028–0.6816]
Mean Calibration	1.24	1.40	1.16	1.30	1.03	1.14	1.12	1.26
Cut-off for high risk* (%)	10.97	9.73	10.18	9.09	9.59	8.7	6.77	6.04
Sensitivity (%)	67	71	71	76	63	70	73	76
Specificity (%)	54	47	52	43	56	48	52	43
Observed risk (%)	15.5	12.5	14.6	11.8	13.7	11.3	10.3	7.9

CR: adjusted for competing non-stroke deaths FSRP: Framingham Stroke Risk Profile. PCE: Pooled Cohort Equations. TIA: Transient Ischemic Attack.

*Global estimate of failure: Kaplan-Meier method for main validation and cumulative incidence function for CR.

### 3.1 FSRP validation

**Fig. 1:**
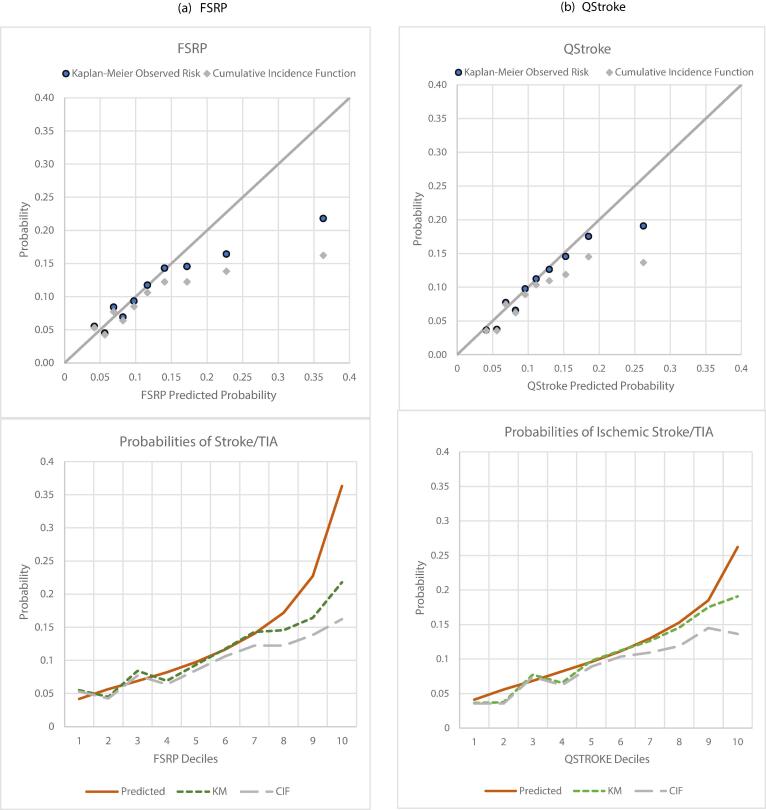

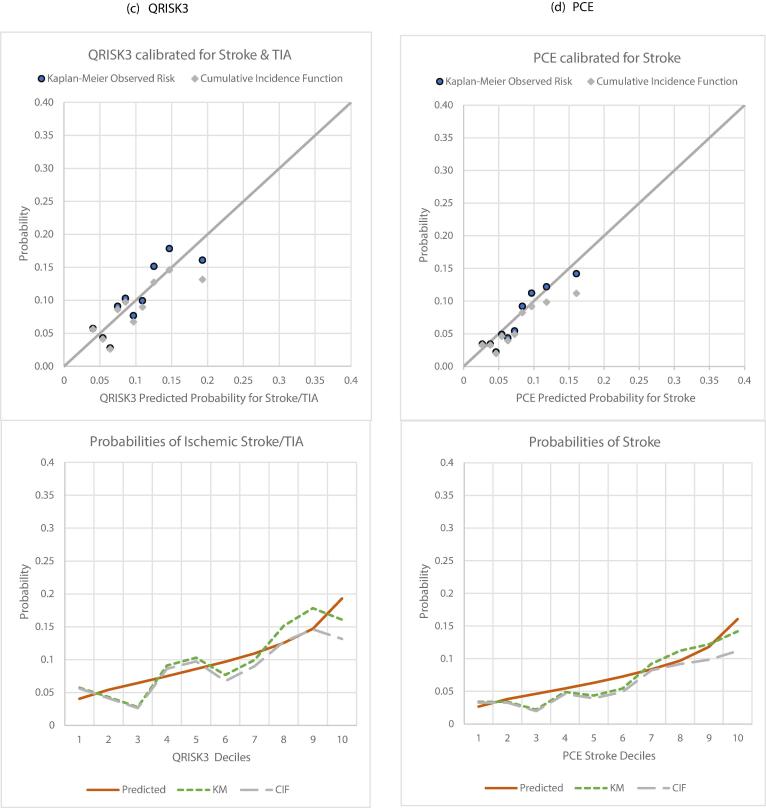
Calibration plots of observed vs predicted risk in deciles of predicted risk (a) FSRP, (b) QStroke, (c) QRISK3 & (d) PCE.

Calibration slope was 0.66 [0.52–0.80], p < 0.001, C-index 0.6346 [0.6068–0.6624] (Table 1). KM curves were separated but slightly less so for low/intermediate and high/very high-risk groups (Supp. Fig. 1a). Mean calibration was 1.24. FSRP over-estimated risk in the upper 3 deciles (Fig. 1a), beyond risk of 15 % and above the KM estimate of failure i.e., 10.97 %. Using the latter as a cut off for high risk, FSRP had Sn/Sp of 67/54 %.

### 3.2 Qstroke validation

Calibration slope was 0.88 [0.69–1.08], p = 0.2661. C-index in BRHS 0.6431 [0.6136–0.6726] (Table 1) was less than that reported by QStroke authors (0.866) (Hippisley-Cox et al., 2013). However, KM curves for the four risk groups were spaced out (Supp. Fig. 1b). Mean calibration was 1.16. Decile-based calibration was good (Fig. 1b) except for over-estimation in the top decile (above predicted risk 21 %). Using the KM risk cut-off (10.2 %), QStroke had Sn/Sp 71/52 %.

### 3.3 QRISK3 validation

Calibration slope was 0.82 [0.58–1.06], p = 0.1407, C-index 0.6317 [0.5970–0.6664] (Table 1), with some separation between KM curves (Supp. Fig. 1c). Mean calibration was 1.03. Decile-based plots showed minor disagreements between observed and predicted risks across the probability range (Fig. 1c). Using the KM risk cut-off (9.6 %), QRISK3 calibrated for stroke had Sn/Sp 63/56 %.

### 3.4 PCE validation

Calibration slope was 0.92 [0.68–1.16], p = 0.50. C-index was 0.6606 [0.6215–0.6997] (Table 1), with overlapping KM curves for low and intermediate risk groups (Supp. Fig. 1d). Mean calibration was 1.12. Predicted probabilities followed the KM failure function closely with slight over estimation in intermediate deciles (Fig. 1d). Using the KM 6.77 % cut off, PCE had Sn/Sp 73/52 %.

Using the National Institute For Health And Care Excellence (10 % (National Institute for Health and Care Excellence, 2014)) and American College of Cardiology/American Heart Association (7.5 % (Arnett et al., 2019, Goff et al., 2014a)) CVD intervention thresholds to respectively categorise men as high or low risk based on QRISK3 and PCE composite-CVD probabilities (prior to correction for stroke outcomes), gave 99 % sensitivity for respective stroke events, with specificity 2–3 % indicating a very high false positive rate. Examining higher cut-offs (Supp. Table 8) improved specificity and positive predictive values at the expense of sensitivity but negative predictive values remained high.

### 3.5 Sensitivity analyses adjusting for competing risks

Adjustment for non-stroke deaths generally worsened discrimination and calibration of all tools (Table 1). Calibration slope deviated further below 1, and when estimated with respect to cumulative incidence function (CIF) of events, mean calibration showed slightly increased global over-prediction. In decile-based plots overestimation was exaggerated (Fig. 1a-d, grey diamonds and dashed line graphs).

### 3.6 Performance of FSRP, QStroke and QRISK3 on a common CVD-free sample

There were 2441 men (mean age 68y) experiencing 113 ischemic strokes and 83 TIA events over 10y (Supp. Tables 9–10). QStroke had a higher C-index 0.6584 [0.6220–0.6949] than FSRP and QRISK3 however confidence intervals for all tools overlapped, and KM survival curves indicated similar discrimination across the 3 tools with FSRP and QStroke discriminating less between low- and intermediate- risk groups (Supp. Fig. 2).

**Fig. 2:**
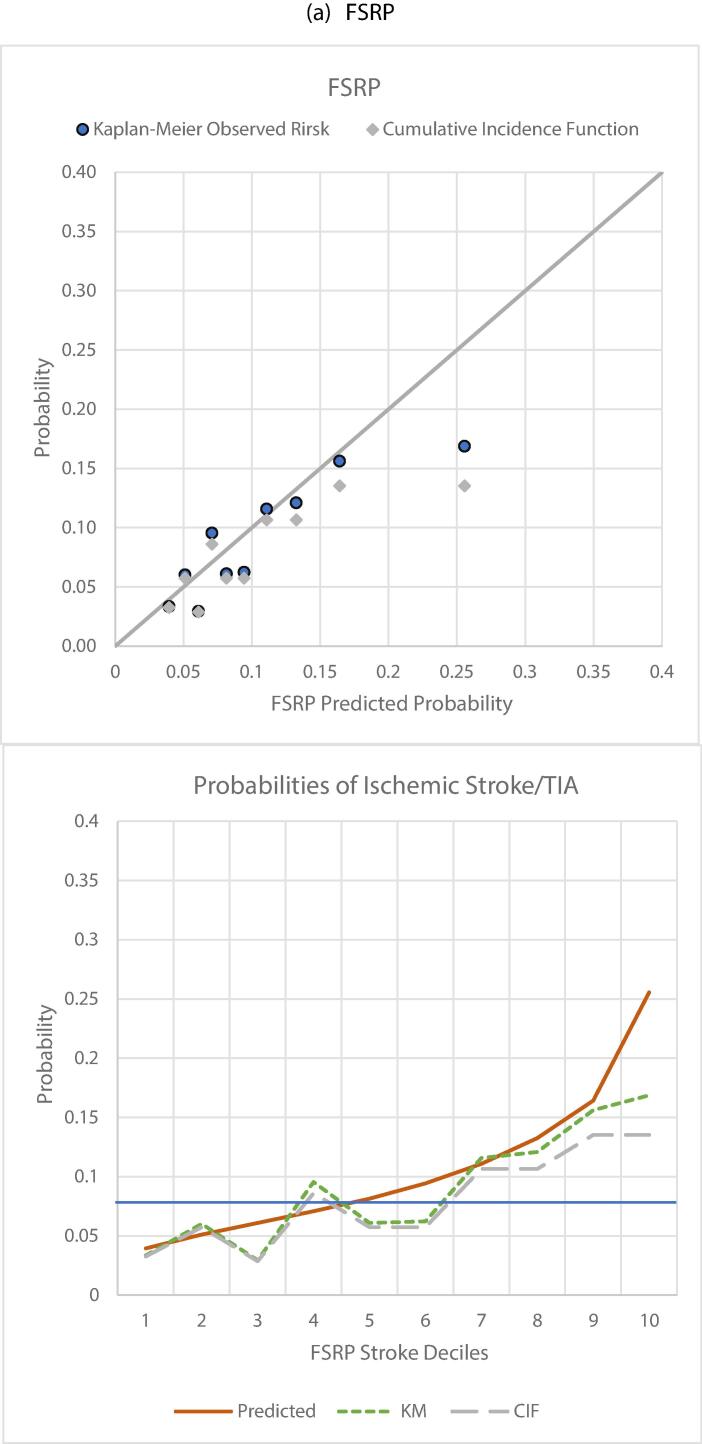
Calibration in a common BRHS sample of 2441 men without CVD, experiencing 196 incident stroke and TIA events over 10y.

**Fig. 3:**
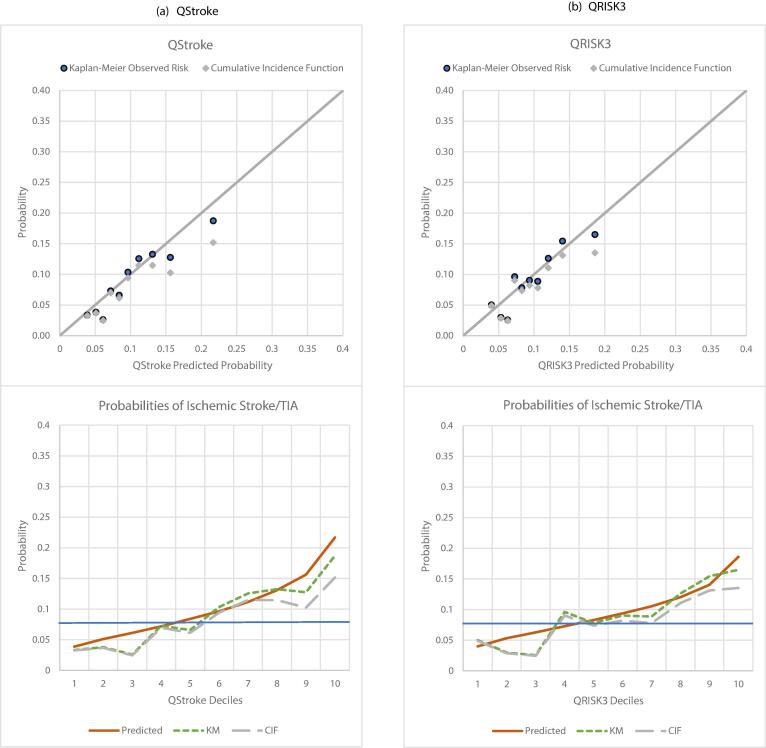
Scatterplot matrix of the FSRP, QStroke and QRISK3 mean predicted probabilities in a common BRHS sample of 2441 men without CVD, experiencing 196 incident stroke and TIA events over 10y.

QRISK3 showed better mean (Supp. Table 10) and decile-based calibration (Fig. 2a-c). Relative overestimation by FSRP and QStroke was more evident in higher deciles particularly with respect to CIF. The tools showed similar Sn/Sp when examined using KM risk and CIF cut-offs. On comparing predicted risks of the three tools according to deciles of their averaged risk, agreement was evident for nearly all except the highest deciles (Fig. 3).

**Fig. 4:**
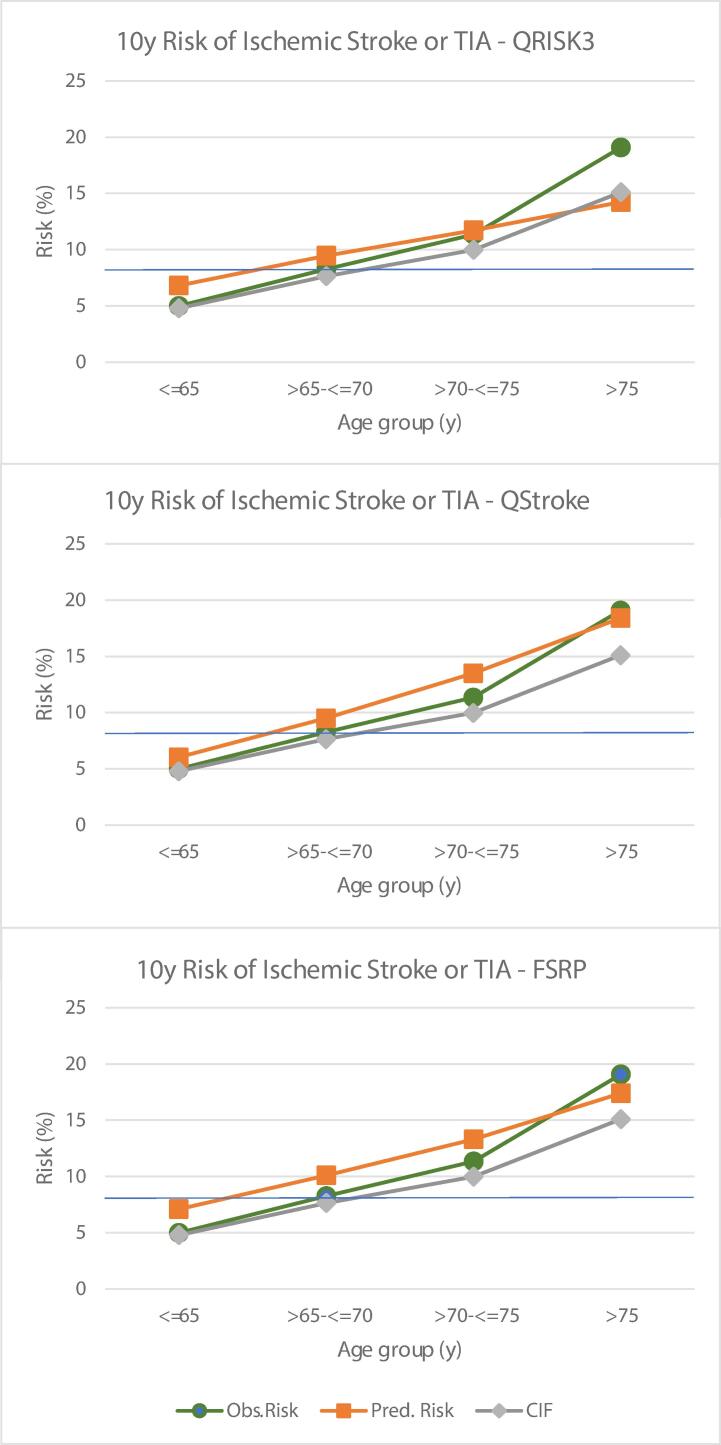
Calibration of QRISK3, QStroke and FSRP across age-groups in 2441 BRHS men without CVD, experiencing 196 incident stroke and TIA events over 10y.

In analysis by age- groups, the gap between CIF and KM risk progressively widened with age (Supp. Tables 11, Fig. 4); for men > 75y, the CIF was 4 % lower than KM risk. Both FSRP and QStroke overestimated risks up to 75y. For men > 75y, mean risk predicted by FSRP was lower than the KM-estimate but higher than the CIF, while that predicted by QStroke was similar to KM-risk and 3 % higher than CIF. QRISK3 predictions were also higher relative to KM and CIF risks, but the difference became smaller with age. In men > 75y, QRISK3 underestimated risk markedly in comparison to the KM estimate, but mean prediction was more aligned with CIF.

## 4 Discussion

With more adults reaching old age, it is necessary to employ the right tool for assessing absolute stroke risk. We investigated how well composite-CVD risk prediction tools QRISK3 and PCE; and stroke-specific FSRP and QStroke captured the true risk of stroke events in older men. Bearing in mind the slightly different stroke outcomes of these tools, we discuss implications of three main findings.

Firstly, both types of tools discriminated only modestly, with discrimination falling further on adjustment for competing risks.

Secondly, stroke-specific FSRP and QStroke tended to overestimate risk at higher predicted probabilities while composite-CVD tools QRISK3 and PCE showed better global calibration with minor mis-estimations across the range of risk. Calibration generally worsened when non-stroke deaths were accounted for. However, in men > 70y without a broad range of cardio/cerebro/vascular conditions, QRISK3 showed better calibration despite adjustment for competing deaths.

Finally, all tools displayed similar sensitivity (63–73 %) and specificity (52–54 %) using validation sample-based observed risk as cut-offs. However, when QRISK3 and PCE were evaluated using risk thresholds recommended for primary prevention of CVD, both falsely categorised a large proportion of men as high risk for stroke events.

### 4.1 Stroke discrimination in older adults needs improvement

The low discrimination of these tools is somewhat expected because of the less heterogenous case-mix of BRHS men (Steyerberg, 2019), particularly with regards to age, the main driver of risk. Comparing stroke-specific scores, predictors like body mass index, cholesterol:HDL ratio, family history of CHD and chronic kidney disease, not part of the FSRP model, help QStroke discriminate ischemic strokes and TIAs marginally better. However, the same additional variables do not seem to improve discrimination as part of QRISK3 that was developed to predict composite risk. This suggests that newer markers being explored for improving CVD risk stratification in older adults should be tested for competing risks adjusted stroke-specific prediction. Coronary artery calcium is one such biomarker which has shown promise for improving risk stratification of CHD but not similarly for stroke (Yano et al., 2017). Until new evidence translates into guidelines, clinical judgement on the use of blood biomarkers associated with stroke risk (Folsom et al., 2013) such as natriuretic peptides which reflect subclinical cardiac dysfunction, and vascular imaging to capture atherosclerotic burden may be helpful on a case-by-case basis (Bambrick et al., 2016).

### 4.2 In older men risk prediction by composite-CVD tools is comparable to if not better than by stroke-specific tools

It is suggested that predicted risk – hence calibration may be more important for clinical decisions than discrimination (Cook, 2007) especially in older populations whose risk distribution is narrower. In men overall, FSRP tended to over-predict risk of stroke/TIA at higher probabilities but estimated risk well across low-mid deciles. This could be because although BRHS men were similar in age to FSRP men, with similar mean PI (D'Agostino et al., 1994, Wolf et al., 1991), they were more frequently using blood pressure medication and fewer of them smoked. Calibration was also good for QStroke – possibly due to having been developed from a UK population that was more contemporaneous to this BRHS sample, except for over-estimation in the highest risk group. In comparison, QRISK3 and even PCE, developed using North American cohorts, displayed slight misestimation through the low-intermediate risk range.

Considering intervention decisions are often made at intermediate risk ranges, the over-prediction by stroke-specific tools in higher deciles may not be of consequence (Nguyen et al., 2020). However, adjustment for non-stroke mortality as a competing event worsened calibration. And in general, CIF diverged more from KM risk with increasing predicted risks. This magnified overestimation which also became apparent at lower predicted risks.

Additionally, both predicted risks and competing mortality increase with ageing (Wolbers et al., 2009). Accordingly, when comparing prediction of ischemic strokes and TIAs in men without CVD, overestimation relative to CIF by stroke specific tools was clearly evident in successive age groups. Yet, QRISK3 showed better calibration in those > 70y. Interestingly, this contrasts with recent findings regarding the effect of non-CVD mortality on the performance of QRISK3 with respect to a composite outcome (Livingstone et al., 2021). We acknowledge our sample is much smaller in comparison but draw attention to the possibility that predictions of composite-CVD and individual components may be operating differently in older populations.

The effect any of this has on clinical utility would depend on the cut-off for intervention. There are no agreed thresholds for stroke risk alone. When we examined the 8 % CIF in CVD-free men (solid blue cut-off Fig. 2, Fig. 4), FSRP appeared more likely than QSTROKE and QRISK3 to misclassify low-risk men (intermediate deciles) as being at high risk, although classification across age bands was comparable.

### 4.3 So, what does this mean for current clinical practice?

Older adults with clinically manifest CHD, HF, arrhythmias and intermittent claudication are generally in receipt of preventive therapies to reduce future CVD events, including strokes. Hence the need for risk stratification becomes more relevant for those ageing without a history of these conditions in whom clinical decisions on interventions are a challenge. In this context, composite-CVD tools like QRISK3 and PCE, developed to aid primary prevention using cohorts that exclude most CVD conditions appear more appropriate for stroke risk prediction. QRISK3 has been recommended for use in adults up to 84y (National Institute for Health and Care Excellence, 2014), and PCE up to 75y (Arnett et al., 2019). While we show that these tools may be reasonably well calibrated for stroke events beyond midlife, using established CVD intervention thresholds of QRISK3 and PCE in older men results in excellent sensitivity but a very high false positive rate for stroke outcomes. This suggests that men may be considered eligible for interventions that they don’t need/benefit from.

Arguably some of these men may be at high risk of CHD when a pharmaceutical intervention such as statin is justified. However, strokes comprise an increasing proportion of first CVD events with increasing age (British Heart Foundation, 2022); and the benefit of statins for the primary prevention of stroke in older adults is debatable for a number of reasons (Saeed, 2020, Shepherd et al., 2002, Volpe and Patrono, 2021). In BRHS, the fraction of hard CVD occurring over 20y that were strokes increased from 30 % when men were followed from a baseline age of 50-70y; to 50 % when followed from 70 to 90y (data not shown).

Moreover, similarly poor specificity has been observed even for broader CVD outcomes when evaluating 7.5 % PCE risk in 66-75y participants of the Framingham Offspring Study, indicating the need for selecting intervention thresholds based on age (Navar-Boggan et al., 2015). Revised European guidelines on CVD prevention take this into consideration and recommend age-specific thresholds (Carballo et al., 2022).

The importance of context in applying risk tools has also been highlighted elsewhere (Gulati et al., 2022, Shah et al., 2022). The implication is that for apparently healthy adults 60y and older, composite-CVD risk models can be used for stroke risk prediction but perhaps need to be (1) updated to reflect their stroke risk more closely; and (2) re-evaluated to ascertain thresholds appropriate for increasing age (Nanna et al., 2020), including for stroke specific work-up/interventions besides statins. Until then, clinicians should be aware of the potential of misclassification and the ever-continuing need for patient discussions on risk enhancers/modifiers and shared decision making.

### 4.4 Limitations

There are some key limitations to our analyses. First, although haemorrhagic strokes have been excluded from models predicting ischemic strokes and TIAs, based on mortality and validation of primary care data; we cannot be sure that this captured all cases of cerebrovascular bleeds as BRHS linkage to hospital episodes is still in progress. However, because of their higher mortality, it is likely that this would be a small number. Second, TIAs have been based on primary care reports according to a clinical, time-based criteria. This may have included TIA mimics. However, TIAs present less frequently to hospital; even within the QRISK development data, majority of TIAs were identified only through primary care records (Hippisley-Cox et al., 2017).

Third, the two Q-models have predictors some of which were not available in BRHS. This includes Townsend scores and type 1 diabetes. However, the alternative index of multiple deprivation used in BRHS was not associated with strokes/TIAs in the sample. And based on self-reported use of insulin only up to 35 men could potentially be type 1 diabetic. BRHS also did not have echocardiographic measures nor direct inquiry on valvular heart disease. But some of these men may have already been excluded by proxy use of anticoagulants per the QStroke model. Systemic lupus erythematosus and mental illness could not be determined, and other predictors like steroid use and erectile dysfunction were reported by few men so it is unclear to what extent they would have contributed to performance regarding stroke. Others have pointed out though that complex models do not necessarily have an advantage over simpler ones (Dziopa et al., 2022). This was in fact here indicated by PCE, based on a handful of core predictors discriminating somewhat better than other tools – and perhaps relates to PCE predicting a more definite outcome of hard strokes only.

We also acknowledge that some comparisons between model performances are based on subjective observation of calibration plots, but (non-test based) visual judgement on calibration to determine the better model is widely used (Collins and Altman, 2012, Schneider et al., 2022, Yourman et al., 2012).

Still, this comparison of four risk tools with regards to stroke prediction has been conducted in a reasonably large sample of older men with near complete 10y follow up. Our findings, particularly those relating to CVD-free men are worth verifying in a larger, multi-ethnic, mixed-gender primary prevention cohort.

### 4.5 Conclusion

In older British men, both stroke-specific and composite-CVD risk tools discriminate stroke risk weakly. Non-stroke deaths influence accuracy of predicted risks, but intervention thresholds determine if competing events are strong enough to limit use of tools. In those without a history of CVD or statins, QRISK3 remains relatively well calibrated for stroke events. However, existing models and/or thresholds should be re-examined to reflect proportional stroke burden in older adults.

## 5 Sources of Funding

AA is funded by UK Medical Research Council Doctoral Training Programme (MR/N013867/1). SPP by UK Medical Research Council Career Development Award (MR/P020372/1). The BRHS is funded by a British Heart Foundation grant (RG/19/4/34452).

The funding bodies had no role in conception, analysis or reporting of this validation work.

## 6 Data Availability

Data supporting the findings of this study are available from the study manager (Ms L Lennon; l.lennon@ucl.ac.uk) upon reasonable request.

## CRediT authorship contribution statement

**Ayesha Ahmed:** Conceptualization, Formal analysis, Writing – original draft, Writing – review & editing. **Gareth Ambler:** Methodology, Supervision, Writing – review & editing. **Snehal M. Pinto Pereira:** Supervision, Writing – review & editing. **Lucy Lennon:** Data curation, Project administration. **Olia Papacosta:** Data curation, Software. **Peter Whincup:** Funding acquisition, Writing – review & editing. **Goya Wannamethee:** Funding acquisition, Methodology, Supervision, Writing – review & editing.

## Declaration of Competing Interest

The authors declare that they have no known competing financial interests or personal relationships that could have appeared to influence the work reported in this paper.

## Data Availability

Data supporting the findings are available from the study manager (Ms L Lennon; l.lennon@ucl.ac.uk) upon reasonable request.
