# Supplementary material for: Stroke risk in older British men: Comparing performance of stroke-specific and composite-CVD risk prediction tools
Source: Prev Med Rep. 2022 Dec 24;31:102098. doi: 10.1016/j.pmedr.2022.102098 (PMC9938339; doi:10.1016/j.pmedr.2022.102098)
Supplement: Supplementary data 1 [file mmc1.docx]

**Supplementary Methods**

1. A prognostic index (PI) was calculated using the published predictor coefficients of each risk tool and BRHS values of predictor variables. For FSRP and PCE, the PI was centred using the published mean PI. For QStroke and QRISK3, continuous variables were centred using published variable means before calculating the PI as done in the available algorithms.
2. A slope of the centred-PI (CPI) was estimated using the CPI as a single predictor in a Cox model. Miscalibration was further tested using a LRtest for beta of the CPI = 1.
3. Proportional Hazards assumption using CPI in the Cox model was tested using the Stata command estat phtest and visually by plots of Schoenfeld residuals against time.
4. Discrimination was assessed using the CPI in a Cox Model and estimating Harrell’s C with 95% confidence intervals using the somersd package. Discrimination was also assessed categorising the CPI according to 16th 50th and 84th centiles and visually comparing KM-survival curves.
5. Predicted probability of the specific stroke outcome(s) was calculated using

P=1-BaselineS(10) ^ exp(CPI)

where BaselineS(10) was the published baseline survivor function at 10y extracted from the relevant risk score.

1. Calibration

*Mean calibration- in the large* – assessed as a ratio of the global expected mean to observed KM probability for each score.

*Moderate calibration*- *decile or age-group based* comparisons of mean predicted probability and group KM failure function at 10y, using pmcalplot package.

1. Sensitivity and Specificity of selected risk cut-offs of the overall KM observed risk (or the conventional composite CVD risk thresholds for intervention 10% for QRISK3 and 7.5% for PCE (1, 2)) were assessed using the stroccurve package to account for censoring.
2. We also examined performance while accounting for competing non-stroke mortality. We used the Fine and Grey (stcrreg) method to get the slope of the CPI and tested if this was different from 1 using Wald test. We followed Wolber’s et al (3) for calculating the C-index for discrimination by adjusting the survival time of men experiencing non-stroke death to greater than the prediction time horizon (>10y). We used the stcompet package to obtain overall and group based cumulative incidence function to assess mean and moderate calibration. Sensitivity and specificity while accounting for competing risks was estimated using stroccurve with adjusted survival time.

**List of Abbreviations**

AF atrial fibrillation

BMI body mass index

BP blood pressure

BRHS British Regional Heart Study

CHD coronary heart disease

CIF cumulative incidence function

CKD chronic kidney disease

CPI centred prognostic index

CVD cardiovascular disease

FSRP Framingham stroke risk profile

HF heart failure

IC intermittent claudication

KM Kaplan-Meier

LVH left ventricular hypertrophy

MI myocardial infarction

NICE National Institute For Health And Care Excellence

PCE pooled cohort equations

PI prognostic index

RA rheumatoid arthritis

SCORE systematic coronary risk evaluation

SLE systemic lupus erythematosus

Sn/SP percent sensitivity/percent specificity

TIA transient ischemic attack

**Table 1: Predictors and outcome event definitions in stroke-specific risk tools and BRHS**

|  | **FSRP (4)** | **QStroke (5)** | **BRHS (6, 7)** |
| --- | --- | --- | --- |
| **Outcome Event** | All types of fatal and non-fatal strokes and TIA | Ischemic fatal and non-fatal strokes and TIA based on Read codes in EHR and ICD10 codes in ONS linked data. | Matched. Mortality ascertained through NHS Central Registers, with cause of death coded using ICD9 430-438. Incident non-fatal events ascertained from ongoing GP reports and 2-yearly primary care record reviews of all surviving participants. GPs identified events on a standard form according to set case-criteria (6, 7). Non-fatal stroke defined as an event producing a neurological deficit of vascular origin for >24h (including subarachnoid haemorrhage, cerebral haemorrhage or thrombosis). In such cases, GPs also asked for information on clinical presentation, hospital summaries and brain scans where available. A BRHS clinical assessor reviewed this material to exclude non-stroke diagnoses. GPs identified a definite TIA as a disturbance of neurological function of presumed vascular origin lasting <24hrs and leaving no residual deficit. |
| **Exclusions** | Prevalent stroke (and TIA) | Prevalent stroke, TIA and use of anticoagulant medication | Matched. Anticoagulants identified by self-reported use of warfarin BNF 2.8.2 |
| **Age (y)** | Self-report in Questionnaire | Self-report in Questionnaire | Self-report in Questionnaire |
| **Townsend Score (continuous)** | - | Postcode linked (Output Area level), 2001 census data. | Not available for 20y re-examination. **(Excluded)** |
| **Ethnicity (categorical)** | - | Self-assigned in EHR. | Questionnaire based, self-reported. Limited to White for this analysis.  **(Excluded)** |
| **Atrial Fibrillation (AF-Y/N)** | By ECG | As recorded in EHR before start of study. | From baseline ECG and GP records |
| **Family h/o of premature coronary heart disease (Y/N)** | - | EHR showing coronary disease in first degree relative less than 60y. | Self-report of a parent’s death from heart disease before age 60y. |
| **Type 1 Diabetes (Y/N)** | - | As recorded in EHR | Not specifically asked - those taking only insulin and so could potentially be type1 are 35 men only),  **(Excluded)** |
| **Type 2 Diabetes (Y/N)** | - | As recorded in EHR | Not specifically asked  **(Coefficient used for any Diabetes)** |
| **Diabetes (Y/N)** | Treatment with insulin or oral medication, or random glucose ≥150mg/dl on 2 or more occasions | - | Self-report of physician diagnosis or diabetic medication or fasting glucose >7 mmol/l |
| **Rheumatoid Arthritis (RA) (Y/N)** | - | As recorded in EHR | Based on self-reported medication linked to selected BNF drug codes 10.1.2.1, 10.1.2.2, 10.1.3,  and 10.1.5. Plus, reason for taking medication coded to ICD9 714.xx |
| **Chronic Kidney Disease (CKD) (Y/N)** | - | As recorded in EHR | eGFR < 60 ml/min per 1.73msq |
| **Hypertension Treatment (Y/N)** | Self-report | EHR diagnosis of hypertension and ≥1 concurrent prescription of ≥1 BP medication | Self-report of selected BNF BP medication codes 2.2.1, 2.2.8, 2.4, 2.5, 2.6.2 |
| **Coronary Heart Disease (CHD-Y/N)** | MI, Angina, Coronary Insufficiency as per Framingham criteria (8) | As recorded in EHR | Self-report of doctor diagnosed/GP record-based MI, Angina, CABG or PTCA |
| **Heart Failure (HF-Y/N)** | As per Framingham criteria (8) | As recorded in EHR | Self-report of doctor diagnosed/GP record-based HF |
| **Intermittent Claudication (IC-Y/N)** | As per Framingham criteria (8) | - | Self-report of doctor-diagnosed or Edinburgh Claudication Questionnaire based IC (9) |
| **Cardiovascular Disease** | CHD, HF and IC | - | Matched |
| **Valvular Heart Disease** | - | As recorded in EHR | Not measured directly (Use of anticoagulants indicated some men with valvular disease, but these get excluded) **(Excluded)** |
| **Body Mass Index (BMI) (kg/m^2^)** | - | EHR value closest to study entry. | From weight and height examined at baseline |
| **Total Cholesterol: HDL Chol Ratio** | - | EHR value closest to study entry. | Fasting blood sample at baseline |
| **Cigarette Smoking** | Self-report – yes/no | EHR value closest to study entry. Ordinal variable (non-, ex-, light-, moderate-, heavy- smoker) | Self-reported at baseline (derived as both binary and ordinal to match definitions) |
| **Systolic BP (mmHg)** | Average of two measurements on clinic visit | EHR value closest to study entry. | Mean of two seated readings at baseline examination |
| **Left Ventricular Hypertrophy (Y/N)** | ECG-based - definite | - | ECG-based - definite |

BRHS: British Regional Heart Study, EHR: electronic health record, ONS: office of national statistics, Y/N = Yes/No

**Table 2: Predictors and outcome event definitions in composite CVD risk tools and BRHS**

|  | **PCE (10)** | **QRISK3 (11)** | **BRHS (6, 7)** |
| --- | --- | --- | --- |
| **Outcome Event** | First hard ASCVD – coronary death or fatal stroke or non-fatal MI or stroke. | Coronary Heart Disease (angina, acute MI, subsequent MI, complications after MI, other acute IHD, chronic IHD), Ischemic Stroke and TIA based on Read codes in GP records, and ICD9/10 codes in HES and ONS linked data. | Matched. Events identified as in Table xx. |
| **Exclusions** | Prevalent MI, stroke, congestive heart failure, percutaneous coronary intervention, coronary bypass surgery and AF and those older than 79 because of complex age interactions. | Prevalent CVD (heart attack, angina, stroke or TIA) & use of statins | Matched |
| **Age (y)** | From ARIC, CHS, CARDIA and FR-Original and Offspring cohorts | From GP record at study entry | Self-report in Questionnaire |
| **Townsend Score (continuous)** | - | Postcode from GP record linked (Output Area level) to 2011 census data. | Not available for 20y re-examination. **(Set to 0/excluded)** |
| **Ethnicity (categorical)** | - | Self-assigned in EHR. | Questionnaire based, self-reported. Limited to White for this analysis.  **(Set to 0/excluded)** |
| **Atrial Fibrillation (AF-Y/N)** | - | Atrial fibrillation, atrial flutter and paroxysmal atrial fibrillation recorded in EHR before start of study. | From baseline ECG and GP records |
| **Family h/o of premature coronary heart disease (Y/N)** | - | EHR showing coronary disease in first degree relative less than 60y. | Self-report of a parent’s death from heart disease before 60y. |
| **Type 1 Diabetes (Y/N)** | - | As recorded in EHR | Not specifically asked  **(Set to 0/excluded)** |
| **Type 2 Diabetes (Y/N)** | - | As recorded in EHR | Not specifically asked  **(Coefficient used for any Diabetes)** |
| **Diabetes (Y/N)** | Cohort specific |  | Self-report of physician diagnosis or diabetic medication or fasting glucose >7 mmol/l |
| **Rheumatoid Arthritis (RA) (Y/N)** | - | RA, Felty’s syndrome, Caplan’s syndrome, adult-onset Still’s disease, inflammatory polyarthropathy not otherwise specified - recorded in EHR | Based on self-reported medication linked to selected BNF drug codes 10.1.2.1, 10.1.2.2, 10.1.3,  and 10.1.5. Reason for taking coded to ICD9 code 714.xx |
| **Chronic Kidney Disease (CKD) (Y/N)** | - | Stage 3-5 CKD, nephrotic syndrome, chronic glomerulonephritis, chronic pyelonephritis, renal dialysis, renal transplant as recorded in EHR | eGFR < 60 ml/min per 1.73msq |
| **Hypertension Treatment (Y/N)** | Cohort specific | EHR diagnosis of hypertension and concurrent prescription of ≥1 BP medication | Self-report of selected BNF BP medication codes 2.2.1, 2.2.8, 2.4, 2.5, 2.6.2 |
| **Migraine** | - | Classic/atypical/abdominal migraine, cluster headache, basilar migraine, hemiplegic migraine and migraine with or without aura | Self-reported medication. BNF code 4.7.4. Reason for taking coded to ICD9 code 346 |
| **Corticosteroid use** | - | BNF Chapter6.3.2 (oral or parenteral) | Self-reported. BNF code 6.3.2 |
| **Atypical antipsychotic use** | - | Second generation antipsychotics | Self-reported. BNF code 4.2.1 |
| **Systemic Lupus Erythematosus (SLE)** | - | SLE, Disseminated Lupus, Libman-Sacks disease | Self-reported medication reason for taking coded to ICD9 code 710.0 |
| **Severe Mental Illness** | - | Psychosis, schizophrenia, bipolar affective disorder | Self-reported medication reason for taking coded to ICD9 code 295, 296, 298. |
| **Erectile dysfunction or treatment** | - | Diagnosis or treatment BNF 7.4.5 | Self-reported. BNF code 7.4.5, Reason for taking coded to ICD9 code 607.84 |
| **Cigarette Smoking** | Self-report – yes/no | EHR value most recent before study entry. Ordinal variable (non-, ex-, light-, moderate-, heavy- smoker) | Self-reported at baseline (derived as both binary and ordinal to match definitions) |
| **BMI (kg/m^2^)** | - | EHR value most recent before study entry. | From weight and height examined at baseline |
| **Total Cholesterol: HDL Chol Ratio or separate** | TC and HDL measures from cohorts | EHR value closest to study entry, restricting those after entry date to ones that were before CVD diagnosis, statin prescription or censoring. | Fasting blood sample at baseline examination |
| **Systolic BP (SBP, mmHg)** | Cohort specific | EHR value most recent before study entry. | Mean of two seated readings at baseline examination |
| **Variability in SBP** | - | SD from all SBP values recorded 5y before study entry | - |

BRHS: British Regional Heart Study, EHR: electronic health record, ONS: office of national statistics, Y/N = Yes/No. PCE: Pooled cohort equations. QRISK3 Use of drugs at baseline: 2 or more prescriptions, most recent <=28 days before study entry.

**Table 3: Missing data comparisons**

P values are for T-tests for continuous and Chi-square tests for categorical variables

1. **FSRP – missingness is 6% of those free of stroke and TIA**

|  | Men with complete data | | Men with missing data | | P for difference |
| --- | --- | --- | --- | --- | --- |
| Stats | N | Mean (SD) or % | N | Mean (SD) or % |  |
| Age | 3762 | 68.5 (5.5) | 219 | 69.1 (5.5) | 0.1222 |
| SBP | 3762 | 141 (24) | 202 | 140 (21) | 0.5525 |
| Bp meds | 3762 | 32 | 168 | 34 | 0.517 |
| Smoking | 3762 | 13 | 212 | 15 | 0.43 |
| Prev. CVD | 3762 | 26 | 219 | 20 | 0.037 |
| Prev. AF | 3762 | 4 | 219 | <5 | 0.911 |
| Prev. LVH | 3762 | 2 | 208 | <5 | 0.183 |
| Prev. DM | 3762 | 11 | 83 | 13 | 0.562 |
| Incident Stroke/TIA | 3762 | 10 | 219 | 10 | 0.878 |

1. **QSTROKE – missingness is 12% of those free of stroke TIA warfarin use and not having a haemorrhagic stroke**

|  | Men with complete data | | Men with missing data | | P for difference |
| --- | --- | --- | --- | --- | --- |
| Stats | N | Mean (SD) or % | N | Mean (SD) or % |  |
| Age | 3376 | 68.4 (5.5) | 468 | 69.0 (5.4) | 0.041 |
| BMI | 3376 | 26.8 (3.6) | 452 | 27.5 (4.3) | 0.0004 |
| Chol:HDL | 3376 | 4.78 (1.4) | 258 | 4.7 (1.5) | 0.3964 |
| SBP | 3376 | 141 (24) | 451 | 140 (24) | 0.382 |
| Smoking | 3376 | 13 | 461 | 12 | 0.451 |
| Prev. AF | 3376 | 3 | 468 | 3 | 0.898 |
| Prev. HF | 3376 | 2 | 468 | 3 | 0.299 |
| Prev. CHD | 3376 | 21 | 468 | 23 | 0.304 |
| Prev. RA | 3376 | <0.5 | 417 | <5 | 0.772 |
| Prev. CKD | 3376 | 11 | 282 | 11 | 0.89 |
| BP meds | 3376 | 30 | 417 | 37 | 0.006 |
| Prev. DM | 3376 | 11 | 338 | 12 | 0.524 |
| Family h/o CHD | 3376 | 5 | 419 | 7 | 0.263 |
| Incident Stroke/TIA | 3376 | 9 | 468 | 9 | 0.947 |

**(C) QRISK3 – missingness is 12% of those free of stroke TIA CHD statin use and not having a haemorrhagic stroke**

|  | Men with  complete data | | Men with  missing data | | P for difference |
| --- | --- | --- | --- | --- | --- |
| Stats | N | Mean (SD) or % | N | Mean (SD) or % |  |
| Age | 2669 | 68.1 (5.4) | 356 | 68.8 (5.6) | 0.0311 |
| BMI | 2669 | 26.7 (3.6) | 348 | 27.2 (4.2) | 0.0139 |
| Chol:HDL | 2669 | 4.8 (1.4) | 207 | 4.7 (1.5) | 0.2649 |
| SBP | 2669 | 142 (24) | 345 | 141 (23) | 0.501 |
| Smoking | 2669 | 13 | 352 | 12 | 0.444 |
| Prev. AF | 2669 | 3 | 356 | 4 | 0.669 |
| Prev. RA | 2669 | <0.5 | 307 | <5 | 0.821 |
| Prev. CKD | 2669 | 9 | 222 | 9 | 0.934 |
| BP meds | 2669 | 22 | 307 | 24 | 0.266 |
| Prev. DM | 2669 | 10 | 249 | 11 | 0.628 |
| Family h/o CHD | 2669 | 5 | 317 | 5 | 0.971 |
| Corticosteroid use | 2669 | <0.5 | 307 | <5 | 0.557 |
| Antipsychotic use | 2669 | <0.5 | 307 | <5 | 0.894 |
| Migraine | 2669 | <0.5 | 307 | <5 | 0.239 |
| Impotence | 2669 | <0.5 | 307 | <5 | 0.631 |
| Incident Stroke/TIA | 2669 | 9 | 356 | 8 | 0.73 |

1. **PCE – missingness is 10% of those free of stroke and PCE criteria of CVD and AF**

|  | Men with  complete data | | Men with  missing data | | P for difference |
| --- | --- | --- | --- | --- | --- |
| Stats | N | Mean (SD) or % | N | Mean (SD) or % |  |
| Age | 3047 | 68.3 (5.4) | 350 | 68.9 (5.6) | 0.0634 |
| SBP | 3047 | 142 (24) | 335 | 142 (23) | 0.747 |
| Chol | 3047 | 234 (40) | 197 | 239 (45) | 0.105 |
| HDL | 3047 | 51.5 (13) | 176 | 53.7 (15) | 0.0354 |
| Bp meds | 3047 | 25 | 301 | 30 | 0.073 |
| Smoking | 3047 | 13 | 345 | 14 | 0.65 |
| Prev. DM | 3047 | 11 | 235 | 10 | 0.828 |
| Incident Stroke/TIA | 3047 | 6 | 350 | 5 | 0.648 |

**(E) Common CVD free sample used for comparing FSRP, QSTROKE and QRSIK3 - missingness is 12% for predictors of all 3 scores**

|  | Men with complete data | | Men with missing data | | P for difference |
| --- | --- | --- | --- | --- | --- |
| Stats | N | Mean (SD) or % | N | Mean (SD) or % |  |
| Age | 2441 | 67.9 (5.3) | 339 | 68.7 (5.5) | 0.0087 |
| BMI | 2441 | 26.9 (3.5) | 331 | 27.2 (4.1) | 0.0124 |
| Chol:HDL | 2441 | 4.8 (1.4) | 198 | 4.7 (1.5) | 0.382 |
| SBP | 2441 | 142 (24) | 328 | 141 (23) | 0.906 |
| Smoking | 2441 | 13 | 335 | 13 | 0.867 |
| Prev. LVH | 2441 | 1 | 331 | <5 | 0.343 |
| Prev. RA | 2441 | 0.41 | 295 | <5 | 0.856 |
| Prev. CKD | 2441 | 9 | 212 | 9 | 0.874 |
| BP meds | 2441 | 20 | 295 | 24 | 0.142 |
| Prev. DM | 2441 | 10 | 236 | 11 | 0.478 |
| Family h/o CHD | 2441 | 5 | 306 | 5 | 0.936 |
| Corticosteroid use | 2441 | <0.5 | 295 | <5 | 0.623 |
| Antipsychotic use | 2441 | <0.5 | 295 | <5 | 0.975 |
| Migraine | 2441 | <0.5 | 295 | <5 | 0.975 |
| Impotence | 2441 | <0.5 | 295 | <5 | 0.728 |
| Incident Stroke/TIA | 2441 | 8 | 339 | 8 | 0.884 |

**Table 4: External Validation of FSRP: Baseline and performance characteristics in the Framingham and BRHS samples**

|  | **BRHS**  **Men free of stroke and TIA** | | **FSRP**  **Men free**  **of stroke** |
| --- | --- | --- | --- |
|  | **Main**  **Validation** | **Adjusted for**  **Competing Risks** |  |
| Sample Size | 3762 | | 2732 |
| Composite Events | 366` | | 213 |
| All Strokes | 221 | | 162 |
| TIAs | 147 | | 51 |
| Competing Events | NA | 900 | - |
|  | | | |
| Age (y) | 68.5 (5.5), 58-81 | | 65.4, 55-84 |
| Systolic BP (mmHg) | 141 (24.3), 73-233 | | 139.3 |
| BP medication | 31.6 | | 16.1 |
| Diabetes | 11.2 | | 10.6 |
| Smoking | 12.8 | | 33.8 |
| Cardiovascular disease | 26 | | 22.2 |
| Atrial Fibrillation | 3.8 | | 2.8 |
| Left Ventricular Hypertrophy | 1.7 | | 3.5 |
|  | | | |
| Mean Prognostic Index [range] | 5.85 [4.2-8.9] | | 5.68 |
| Slope of Centred Prognostic Index  [95% CI] | 0.66  [0.52-0.80] | 0.55  [0.41-0.68] | NA |
| Harrell’s C  [95% CI] | 0.6346  [0.6068-0.6624] | 0.6155  [0.5876-0.6434] | NA |
| 10y Pred. Probability (%)  Mean [Range]  Median [Interquartile range] | 13.7 [2.28-91.2]  10.6 [6.88, 17.0] | | NA |
| Mean Calibration | 1.24 | 1.40 | NA |
| Cut-off for high risk* (%) | 10.97 | 9.73 | - |
| Sensitivity (%) | 67 | 71 | - |
| Specificity (%) | 54 | 47 | - |
| Observed risk (%) | 15.5 | 12.5 | - |

`2 men had both TIA and Stroke on the same day. *Global estimate of failure: KM for main analysis and cumulative incidence function for competing risks adjustment. Continuous variables show mean (sd), range. The FSRP publication does not provide a measure of SD. Categorical variables show %.

**Table 5: External Validation of QStroke: Baseline and performance characteristics in QResearch and BRHS samples**

|  | **BRHS**  **Men free of stroke, TIA,**  **anticoagulant use** | | **QStroke^**  **Men free of stroke, TIA, anticoagulant use** |
| --- | --- | --- | --- |
|  | **Main**  **Validation** | **Adjusted for**  **Competing Risks** |  |
| Sample Size | 3376 | | 1748108 |
| Composite of *Ischaemic* Stroke or TIA | 307 | | 38074 |
| Competing events | NA | 784 | - |
|  |  |  |  |
| Age (y) | 68 (5.5), 58-81 | | 45 (15.4), 25-84 |
| Townsend Score | **Excluded** | | -0.1 (3.5) |
| Body Mass Index (kg/m^2^) | 26.8 (3.6), 14-48 | | 26.0 (4.6) |
| Systolic BP (mmHg) | 141 (24.2), 73-233 | | 130 (19.9) |
| Total Cholesterol: HDL Cholesterol | 4.8 (1.4), 2-12 | | 4.1 (1.3) |
| Ethnicity | 99 White/not recorded  **Excluded** | | 94 White/  not recorded |
| Atrial Fibrillation | 2.7 | | 0.4 |
| Premature Coronary Heart Disease  in family | 5.4 | | 12.0 |
| Type 1 Diabetes | **Excluded** | | 0.3 |
| Type 2 Diabetes | 11 (any diabetes) | | 2.2 |
| Rheumatoid Arthritis | <0.5 | | 0.6 |
| Chronic Kidney Disease | 11.1 | | 0.2 |
| Hypertension Treatment | 30.3 | | 6.7 |
| CHD | 21 | | 2.8 |
| Heart Failure | 2.2 | | 0.5 |
| Valvular Heart Disease | **Excluded** | | 0.4 |
| Non-smoker | 30 | | 50 |
| Ex-smoker | 57 | | 17 |
| Light smoker | 4 | | 7 |
| Moderate smoker | 5 | | 8 |
| Heavy smoker | 4 | | 6 |
| Current smoker | 13 | | 25 |
| Slope of Centred Prognostic Index  [95% CI] | 0.88  [0.69-1.08] | 0.71  [0.53-0.90] |  |
| Harrell’s C  [95% CI] | 0.6431  [0.6136-0.6726] | 0.6199  [0.5904-0.6495] | 0.866 |
| 10y Predicted Probability (%)  Mean [Range]  Median [Interquartile range] | 11.8 [2.2-58.0]  10.3 [6.8, 15.3] | | NA |
| Mean Calibration | 1.16 | 1.30 | NA |
| Cut-off for high risk* (%) | 10.18 | 9.09 | - |
| Sensitivity (%) | 71 | 76 | - |
| Specificity (%) | 52 | 43 | - |
| Observed risk (%) | 14.6 | 11.8 | - |

*^Baseline characteristics given for total sample – men (49%) & women.* *Global estimate of failure: KM for main analysis and cumulative incidence function for competing risks adjustment. Continuous variables show mean (sd), range. Categorical variables show %.

**Table 6: External Validation of QRISK3 calibrated for stroke events: Baseline and performance characteristics in the QResearch and BRHS samples**

|  | **BRHS**  **Men free of CHD Stroke TIA &**  **Statin use** | | **QRISK3**  **Men free of CVD &**  **Statin use** |
| --- | --- | --- | --- |
|  | **Main**  **Validation** | **Adjusted for**  **Competing Risks** |  |
| Sample Size | 2669 | | 3869847 |
| Composite of *Ischaemic* Stroke or TIA | 232 | | 133097 ALL |
| Competing events | - | 570 |  |
|  | | | |
| Age (y) | 68 (5.4), 58-81 | | 42.6 (14), 25-84 |
| Townsend Score | **Excluded** | | 0.5 (3.3) |
| BMI (kg/m^2^) | 26.7 (3.6), 14-48 | | 25.9 (4.2) |
| Systolic BP (mmHg) | 142 (24), 81-233 | | 129.2 (16.3) |
| SBP Variability | - | | 9.9 (6.8) |
| Total Cholesterol: HDL Cholesterol | 4.8 (1.4), 2-12 | | 4.4 (1.4) |
| Ethnicity | 99 White/not recorded  **Excluded** | | 89 White/  not recorded |
| Atrial Fibrillation | 3.2 | | 0.5 |
| Premature Coronary Heart Disease  in family | 5 | | 9 |
| Type 1 Diabetes | **Excluded** | | 0.3 |
| Type 2 Diabetes | 10.3 | | 1.5 |
| Rheumatoid Arthritis | <0.5 | | 0.5 |
| Chronic Kidney Disease | 9.3 | | 0.3 |
| Hypertension Treatment | 21.7 | | 4.2 |
| Migraine | <0.5 | | 2.7 |
| Corticosteroid use | <0.5 | | 1.5 |
| Atypical antipsychotic use | <0.5 | | 0.5 |
| SLE | **Excluded** | | <0 |
| Severe Mental Illness | **Excluded** | | 4.3 |
| Erectile dysfunction or treatment | <0.5 | | 2.3 |
| Non-smoker | 32 | | 38 |
| Ex-smoker | 56 | | 15 |
| Light smoker | 4 | | 13 |
| Moderate smoker | 5 | | 7 |
| Heavy smoker | 4 | | 5 |
|  |  | |  |
| Slope of Centred Prognostic Index  [95% CI] | 0.82  [0.58-1.06] | 0.69  [0.47-0.92] |  |
| Harrell’s C  [95% CI] | 0.6317  [0.5970-0.6664] | 0.6148  [0.5800-0.6496] | 0.858 |
| 10y Pred. Probability (%)  Mean [Range]  Median [Interquartile range] | 9.91 [2.1-29.9]  9.15 [6.4, 12.5] | | NA |
| Mean Calibration | 1.03 | 1.14 | NA |
| Cut-off for high risk* (%) | 9.59 | 8.7 | - |
| Sensitivity (%) | 63 | 70 | - |
| Specificity (%) | 56 | 48 | - |
| Observed risk (%) | 13.7 | 11.3 | - |

*Global estimate of failure: KM for main analysis and cumulative incidence function for competing risks adjustment. Continuous variables show mean (sd), range. Categorical variables show %.

**Table 7: External Validation of PCE calibrated for stroke events: Baseline and performance characteristics in the PCE and BRHS samples**

|  | **BRHS Men free of Stroke, MI, HF & AF** | | **Men from PCE Cohorts free of Stroke, MI, HF & AF** |
| --- | --- | --- | --- |
|  | **Main Validation** | **Adjusted for**  **competing risks** |  |
| Sample Size | 3047 | | 9098 White Men |
| Events | Strokes (TIAs not included) 184 | | All CVD: 1259 |
| Competing events | NA | 673 | - |
|  | | | |
| Age (y) | 68 (5.4), 58-81 | | 56.1 |
| Untreated SBP (mmHg) | 140 (24), 81-233 | | 120 |
| Treated SBP (mmHg) | 150 (23), 83-232 | | 136 |
| Systolic BP (mmHg) | 142 (24), 81-233 | | - |
| Total Chol (mg/dl) | 234 (40), 116-448 | | 210.7 |
| HDL Chol (mg/dl) | 52 (13), 19-127 | | 44.4 |
| BP meds | 25.2 | | 16.8 |
| Diabetes | 10.7 | | 8.75 |
| Smoking | 13.3 | | 25.4 |
|  |  | |  |
| Mean Prognostic Index [range] | 62.24 [60.38-64.26] | | 61.18 |
| Slope of Centred Prognostic Index  [95% CI] | 0.92  [0.68-1.16] | 0.79  [0.56-1.02] |  |
| Harrell’s C  [95% CI] | 0.6606  [0.6215-0.6997] | 0.6422  [0.6028-0.6816] | 0.7443 |
| 10y Pred. Probability (%)  Mean [Range]  Median [Interquartile range] | 7.60 [1.2-25.7]  6.74 [4.6, 9.6] | | NA |
| Mean Calibration | 1.12 | 1.26 | NA |
| Cut-off for high risk* (%) | 6.77 | 6.04 | - |
| Sensitivity (%) | 73 | 76 | - |
| Specificity (%) | 52 | 43 | - |
| Observed risk (%) | 10.3 | 7.9 | - |

*Global estimate of failure: KM for main analysis and cumulative incidence function for competing risks adjustment. Continuous variables show mean (sd), range. Categorical variables show %.

**Table 8: Comparing different cut-offs of composite CVD risk tools for capture of stroke outcomes**

| **Cut-off for high risk (%)** | **Sensitivity (%)** | **Specificity (%)** | **PPV**  **(%)** | **NPV**  **(%)** |
| --- | --- | --- | --- | --- |
| QRISK3 (n=232 events /2669 men) |  |  |  |  |
| 10 | 99 | 3 | 8.8 | 97 |
| 15 | 93 | 17 | 9.5 | 95 |
| 20 | 83 | 35 | 10.7 | 95 |
| 25 | 67 | 52 | 11.3 | 94 |
| 30 | 55 | 68 | 13.0 | 94 |
|  |  |  |  |  |
| PCE (n=184 events/3047 men) |  |  |  |  |
| 7.5 | 99.5 | 2 | 6.1 | 98 |
| 10 | 96 | 8 | 6.2 | 96 |
| 15 | 88 | 24 | 6.9 | 97 |
| 20 | 78 | 43 | 7.9 | 97 |
| 25 | 67 | 59 | 9.11 | 96 |
|  |  |  |  |  |

PPV: positive predictive value. NPV: negative predictive value

**Table 9: Characteristics of BRHS men free of CVD in the common sub-sample used for validating FSRP, QStroke and QRISK3 for the outcome of stroke or TIA**

|  | **BRHS (1998-2000)**  **Men free of Stroke, TIA, CHD, HF, IC, AF, Anticoagulant & Statin use** |
| --- | --- |
|  |  |
| Sample Size | 2441 |
| Composite of *Ischaemic* Stroke or TIA | 196 |
| Ischemic Strokes | 116 |
|  |  |
| Age (y) | 68 (5.3), 58-81 |
| Townsend Score | **Excluded** |
| BMI (kg/m^2^) | 26.7 (3.5), 14-48, |
| SBP (mmHg) | 142 (24), 81-233 |
| Total Cholesterol: HDL Cholesterol | 4.8 (1.4), 2-12 |
| Cholesterol (mmol/l) | 6.09 (1), 3-12 |
| HDL (mmol/l) | 1.34 (0.3), 0.5-3.3 |
| Premature Coronary Heart Disease in family | 5 |
| Type 1 Diabetes | **Excluded** |
| Type 2 Diabetes | 9.6 |
| Rheumatoid Arthritis | <0.5 |
| Chronic Kidney Disease | 8.6 |
| Hypertension Treatment | 20 |
| Left Ventricular Hypertrophy | 1.2 |
| Migraine | <0.5 |
| Corticosteroid use | <0.5 |
| Atypical antipsychotic use | <0.5 |
| SLE | **Excluded** |
| Severe Mental Illness | **Excluded** |
| Erectile dysfunction or treatment | <0.5 |
| Non-smoker | 33 |
| Ex-smoker | 55 |
| Light smoker | 3 |
| Moderate smoker | 5 |
| Heavy smoker | 4 |
| Current smoker | 12.9 |
| Ethnicity | 99 White/not recorded **Excluded** |
| Valvular Heart Disease | **Excluded** |

Continuous variables show mean (sd), range. Categorical variables show %.

**Table 10: External Validation of FSRP, QStroke and QRISK3 in a common BRHS sample of men without CVD (N=2441, mean age 68y),** *adjustment for competing non-stroke deaths in italics.*

|  | **FSRP** | | **QStroke** | | **QRISK3 calibrated for stroke/TIA** | |
| --- | --- | --- | --- | --- | --- | --- |
| **Men free of** | Stroke, TIA, CHD, HF, IC, AF, Anticoagulant use & Statin use | | | | | |
| **Outcome (N)** | Ischemic Strokes (113) and TIA (83) N=196; | | | | | |
| **Competing events (N)** | 474 | | | | | |
|  |  | | | | | |
| **Slope of CPI [95% CI]** | 0.83 [0.60-1.05] | | 1.05 [0.78-1.31] | | 0.90 [0.64-1.17] | |
| **P test for slope CPI=1** | 0.1312 | | 0.7284 | | 0.4629 | |
| *Slope of CPI [95% CI]* | *0.72 [0.50-0.93]* | | *0.91 [0.66-1.16]* | | *0.78 [0.54-1.03]* | |
| *P test for slope CPI=1* | *0.0091* | | *0.4850* | | *0.0852* | |
|  |  | |  | |  | |
| **Harrell’s C**  **[95% CI]** | 0.6400  [0.6016-0.6783] | | 0.6584  [0.6220-0.6949] | | 0.6408  [0.6033-0.6783] | |
| *Harrell’s C [95% CI]*  *Adj. for competing risks* | 0.6267  [0.5884-0.6650] | | 0.6411  [0.6046-0.6777] | | 0.6254  [0.5878-0.6631] | |
|  |  | |  | |  | |
| **10y Pred. Probability (%)**  **Mean [Range]**  **Median [Interquartile range]** | 10.6 [2.3-68.5]  8.77 [6.1-13.2] | | 10.2 [2.12-33.5]  9.06 [6.1, 13.2] | | 9.57 [2.14-29.9]  8.87 [6.26, 12.0] | |
|  |  | |  | |  | |
| **Mean Calibration** | 1.21 | | 1.16 | | 1.09 | |
| *Adj. for competing risks* | 1.32 | | 1.27 | | 1.19 | |
|  |  | |  | |  | |
| **Time-dependent ROC Analysis** | Sens/  Spec | Obs. Risk | Sens/  Spec | Obs. Risk | Sens/  Spec | Obs. Risk |
| KM cut-off 8.79% | 69/52 | 12.4 | 76/51 | 13.3 | 69/51 | 12.2 |
| CI cut-off 8.03% | 72/46 | 10.4 | 78/44 | 10.8 | 75/44 | 10.4 |

CPI: centred prognostic index

**Table 11: Observed and predicted risks among 2441 BRHS men free of CVD, mean age 68y**

| Age group (y) | No. of Events/N | Cumulative Incidence (%) | Kaplan-Meier Risk (%) | FSRP Predicted Risk (%) | QStroke Predicted Risk (%) | QRISK3 Predicted Risk (%) |
| --- | --- | --- | --- | --- | --- | --- |
| <=65 | 42/876 | 4.79 | 4.99 | 7.1 | 6.03 | 6.81 |
| >65 - <=70 | 57/746 | 7.64 | 8.26 | 10.1 | 9.5 | 9.46 |
| >70 - <=75 | 52/521 | 9.98 | 11.34 | 13.3 | 13.54 | 11.7 |
| >75 | 45/298 | 15.1 | 19.08 | 17.4 | 18.4 | 14.2 |

**Fig 1: Kaplan-Meier curves for risk groups based on prognostic index (a) FSRP, (b) QStroke, (c) QRISK3 & (d) PCE**

**(a)**

**(b)**

**(c)**

**(d)**

**Fig 2: Kaplan-Meier curves for risk groups in a common BRHS sample of 2441 men without CVD, experiencing 196 incident stroke and TIA events over 10y**

**References**

1. Arnett DK, Blumenthal RS, Albert MA, Buroker AB, Goldberger ZD, Hahn EJ, et al. 2019 ACC/AHA Guideline on the Primary Prevention of Cardiovascular Disease: Executive Summary: A Report of the American College of Cardiology/American Heart Association Task Force on Clinical Practice Guidelines. Circulation. 2019;140(11):E563-E95.

2. National Institute for Health and Care Excellence. Cardiovascular disease: risk assessment and reduction, including lipid modification. 2014.

3. Wolbers M, Koller MT, Witteman JCM, Steyerberg EW. Prognostic Models With Competing Risks Methods and Application to Coronary Risk Prediction. Epidemiology. 2009;20(4):555-61.

4. D'Agostino RB, Wolf PA, Belanger AJ, Kannel WB. Stroke risk profile: adjustment for antihypertensive medication. The Framingham Study. Stroke. 1994;25(1):40-3.

5. Hippisley-Cox J, Coupland C, Brindle P. Derivation and validation of QStroke score for predicting risk of ischaemic stroke in primary care and comparison with other risk scores: a prospective open cohort study. Bmj. 2013;346:f2573.

6. Lennon LT, Ramsay SE, Papacosta O, Shaper AG, Wannamethee SG, Whincup PH. Cohort Profile Update: The British Regional Heart Study 1978-2014: 35 years follow-up of cardiovascular disease and ageing. International Journal of Epidemiology. 2015;44(3).

7. Walker M, Whincup PH, Shaper AG. The British Regional Heart Study 1975-2004. Int J Epidemiol. 2004;33(6):1185-92.

8. Cupples L. Section 34: some risk factors related to the annual incidence of cardiovascular disease and death in pooled repeated biennial measurements. Framingham Heart Study: 30 Year Follow FollUp. 1987:1-22.

9. Leng GC, Fowkes FG. The Edinburgh Claudication Questionnaire: an improved version of the WHO/Rose Questionnaire for use in epidemiological surveys. J Clin Epidemiol. 1992;45(10):1101-9.

10. Goff DC, Lloyd-Jones DM, Bennett G, Coady S, D'Agostino RB, Gibbons R, et al. 2013 ACC/AHA Guideline on the Assessment of Cardiovascular Risk A Report of the American College of Cardiology/American Heart Association Task Force on Practice Guidelines. Circulation. 2014;129(25):S49-S73.

11. Hippisley-Cox J, Coupland C, Brindle P. Development and validation of QRISK3 risk prediction algorithms to estimate future risk of cardiovascular disease: prospective cohort study. BMJ. 2017;357:j2099.
